# Deletions of conserved extracytoplasmic function sigma factors-encoding genes in *Streptomyces* have a major impact on secondary metabolism

**DOI:** 10.1186/s12934-024-02479-x

**Published:** 2024-07-18

**Authors:** Olga N. Sekurova, Martin Zehl, Michael Predl, Peter Hunyadi, Thomas Rattei, Sergey B. Zotchev

**Affiliations:** 1 Department of Pharmaceutical Sciences, Division of Pharmacognosy, University of Vienna, Vienna, 1090 Austria; 2 Department of Analytical Chemistry, Faculty of Chemistry, University of Vienna, Vienna, 1090 Austria; 3 Centre for Microbiology and Environmental Systems Science, University of Vienna, Vienna, 1030 Austria; 4 Doctoral School in Microbiology and Environmental Science, University of Vienna, Vienna, 1030 Austria

**Keywords:** Streptomyces bacteria, Transcriptomics, Extracytoplasmic function sigma factors, Secondary metabolism

## Abstract

**Background:**

Ethanol shock significantly affects expression of over 1200 genes in *Streptomyces venezuelae* NRRL B-65,442, including those involved in secondary metabolite biosynthesis and a cryptic gene *pepX*, which encodes a 19-amino acid peptide with an unknown function.

**Results:**

To establish a possible correlation between the PepX peptide and secondary metabolism in *S. venezuelae*, its gene was deleted, followed by analyses of the transcriptome and secondary metabolome of the mutant. Although the secondary metabolome of the *pepX* mutant was not strongly affected, *pepX* deletion, similar to ethanol shock, mostly resulted in downregulated expression of secondary metabolite biosynthesis gene clusters (BGCs). At the same time, there was a reverse correlation between the expression of certain extracytoplasmic function sigma factors (ECFs) and several BGCs. Individual deletions of three selected ECF-coding genes conserved in *Streptomyces* that were upregulated upon both *pepX* deletion and ethanol shock, had a profound positive effect on the expression of BGCs, which also correlated with the overproduction of specific secondary metabolites. Deletion of one such ECF-coding gene in a marine sponge-derived *Streptomyces* sp. also significantly altered the secondary metabolite profile, suggesting an important role of this ECF in the regulation of secondary metabolism.

**Conclusions:**

These findings pave the way for the activation or upregulation of BGCs in *Streptomyces* bacteria harboring genes for ECFs homologous to those identified in this study, hereby assisting in the discovery of novel bioactive secondary metabolites.

**Supplementary Information:**

The online version contains supplementary material available at 10.1186/s12934-024-02479-x.

## Background

Most bacteria live in complex environments such as soils, marine sediments, plant and animal hosts, where they constantly encounter multiple stressors in the form of living competitors for nutritional sources, predators, as well as abiotic factors such as shifts in temperature, salinity, pH etc. To cope with these challenges, bacteria have developed several regulatory systems that respond to particular environmental cues and adequately change gene expression patterns to quickly adapt to the new situation. One of such response systems is represented by an array of extracytoplasmic function sigma factors (ECFs) and their cognate anti-sigma factors, whose genes have been identified in all sequenced bacterial genomes [1]. Sigma factors represent the essential parts of the RNA polymerase holoenzyme that bind promoter regions and recruit the rest of the enzyme subunits to the transcription start site. Unlike housekeeping sigma factors, such as σ^70^, which recognize promoters of genes with fundamental roles in bacterial cell functioning (e.g. DNA replication, protein and cell wall syntheses, etc.), ECFs represent the most diverse group of sigma factors that ensure the expression of genes whose products are only needed in particular circumstances. The specificity of ECFs is supported by their ability to recognize only the − 10 and − 35 promoter regions with rather invariable spacing, while housekeeping sigma factors require in addition an upstream and extended − 10 regions to efficiently bind the promoter [2]. Typically, ECFs are bound to anti-ECFs, which are often tethered to the bacterial membrane, and are thus unable to bind promoters. This sequestration of ECFs is relieved upon proteolytic digestion or conformational change of anti-ECFs, which may be caused by, for example, proteases produced by other bacteria or environmental stress affecting membrane integrity [3, 4].

Bacteria of the genus *Streptomyces* are ubiquitous with regard to environments they inhabit and have evolved a large number of ECF-anti-ECF pairs to respond to environmental cues. The genomes of *Streptomyces* bacteria typically encode over 50 sigma factors, most of them being ECFs, some of which are involved in regulation of secondary metabolism. Many secondary metabolites produced by *Streptomyces* are medically important, such as antibacterial, antifungal, antitumor and immunosuppressive agents [5]. Insights into the correlation between environmental stress and secondary metabolite biosynthesis may lead to new strategies for increasing the yields and producing novel compounds whose biosynthesis gene clusters (BGCs) are not expressed in standard conditions. ECFs can play various and complex roles in controlling secondary metabolite biosynthesis. For instance, the biosynthesis of 3-formamidosalicylate, the starter unit for antimycin biosynthesis, is positively regulated by ECF^AntA^ encoded within the antimycin BGC, which is antagonized by the ClpXP-protease system in *Streptomyces albus* S4 [6]. Conversely, Rebets et al. [7] have demonstrated that the deletion of certain ECF-coding genes in *Streptomyces lividans* TK24 results in the overproduction of the polyketide actinorhodin and the siderophore coelichelin [7]. Although in the case of antimycin the dependence of gene expression on a specific sigma factor provides a straightforward explanation for the activator role of the ECF^AntA^, the positive effect of ECF gene deletions on secondary metabolism may involve several possible scenarios. For example, such ECFs may control the expression of negative regulators that suppress particular BGCs, or they may compete for promoter regions of BGC-situated genes with housekeeping sigma factors, thereby preventing the latter from binding [8].

In this study, we investigated the effects of several genes whose expression was affected by ethanol shock in *Streptomyces venezuelae* NRRL B-65,442 by constructing deletion mutants and exploring their transcriptomes and secondary metabolomes. In particular, we examined the effect of deletion of a gene encoding 19-amino acid peptide PepX on secondary metabolome of *S. venezuelae*. The expression of the *pepX* gene was shown in the previous study to be significantly downregulated by ethanol shock, which coincided with the downregulation of certain BGCs [9]. We identified three genes encoding ECF sigma factors conserved in *Streptomyces* bacteria, whose deletions lead to activation of the expression of specific BGCs, prompting a significant increase in the production of certain secondary metabolites. Similar results for at least one such ECF-coding gene were obtained for the non-model marine sponge-derived *Streptomyces* sp. ADI96-15.

## Materials and methods

### Growth conditions, fermentations and ethanol shock

Growth conditions for *Streptomyces venezuelae* NRRL B-65,442 and deletion mutants were as described in Sekurova et al. [9]. All fermentations were performed in triplicates. Methanolic culture extracts were prepared according to Sekurova et al. [9]

### Construction of plasmids and deletion mutants

#### Vector pSOK910

Suicide vector pSOK910 for making gene deletions in the *Streptomyces* chromosomes via homologous recombination was constructed by ligation of synthetic part ermE*p-*gusA* (reporter gene for β-glucoronidase under control of a strong constitutive promoter ermE*p) with 3-kb *Hin*dIII-*Eco*RI fragment from pSOK201 [10].

#### Vector pKNOCK

Suicide vector pKNOCK was used for deletion of gene Sig04314 from the chromosome of *Streptomyces* sp. ADI196-15. It was constructed by ligation of a codon-optimized *sfp* gene (*Bacillus subtilis* phosphopantetheinyl transferase, synthesized at BioCat, Germany) with part of the vector pPCV301 (Figure S1, Supplementary Information) cut with *Hin*dIII + *Xba*I, and containing reporter gene *bpsA* (encodes indigoidine synthetase and responsible for the production of blue pigment indigoidine), *aac* [3]*-IV* gene for apramycin resistance and RP4 origin for conjugative DNA transfer.

#### pepX deletion mutant

The vector pPXD910 for *pepX* gene deletion was constructed by PCR amplification of flanking regions of the *pepX* gene using primers PEXDF1, PEXDR1 (Amplicon 1) and PEXDF2, PEXDR2 (Amplicon 2), Table S1, Supplementary Information). Amplicon 1 was cut with *Eco*RI + *Bam*HI, Amplicon 2 - with *Bam*HI + *Xba*I, and these fragments were ligated together with the vector pSOK910 cut with *Eco*RI + *Xba*I. The final construct pPXD910 was introduced into *S. venezuelae* via conjugation. The first crossover mutant went through 4 passages of growth in liquid TSB medium without antibiotic selection, and was plated on ISP4 medium for sporulation. The spore suspension and its serial dilutions were prepared, and 100 µl of -6 dilution was plated on 10 ISP4 plates containing X-Gluc (5-bromo-4-chloro-3-indolyl-β-D-glucuronide cyclohexylammonium salt). After several days, blue and white colonies appeared on the plates. White clones, which no longer harbor the reporter gene *gusA* (second crossover candidates), were selected. Genomic DNA form these clones were isolated and used as a template for PCR to confirm the deletion of *pepX* gene using primers PepXdel-F and PepXdel-R (Table S1, Supplementary Information).

#### pepX overexpression vector

The *pepX* gene with its native Shine-Dalgarno sequence and an upstream P21 promoter [11] was synthesized at BioCat (Germany) and cloned as *Eco*RI-*Xba*I fragment into the vector pSOK806 [12]. The resulting plasmid designated pPXE1 was introduced into the *S. venezuelae* by conjugation.

#### Construction of the ECF sigma factors-encoding genes deletion mutants in *S. venezuelae*

The vectors pECF200, pECF300 and pECF501 for the in-frame deletions of ECF genes from *S. venezuelae* genome were constructed by PCR amplifications of flanking regions for corresponding genes, using primers ECF2delA-F and ECF2delA-R for flankA, and ECF2delB-F and ECF2delB-R for flank B for ECF200 deletion; primers ECF3delA-F, ECFdelA-R, ECF3delB-F and ECF3delB-R for ECF300 deletion, and ECF5delA-F, ECF5delA-R, ECF5delB-F and ECF5delB-R for ECF500 deletion (Table S1, Supplementary Information). All “A” amplicons were cut with *Pst*I + *Xba*I, all “B” amplicons -with *Pst*I + *Eco*RI. The fragments were then ligated in pairs A + B into pSOK910 vector cut with *Xba*I + *Eco*RI. The final constructs were introduced into *S. venezueale* by conjuagation. First crossover mutants went through several rounds of growth in liquid TSB medium without antibiotic selection, and were plated for sporulation on ISP2 medium. Serial dilutions of spore suspension were prepared, and − 5 dilution plated on 10 ISP4 plates with X-Gluc. After several days, white and blue colonies appeared on the plates, and genomic DNAs from selected white colonies, as the candidates for second crossover mutants, were extracted. The following PCR with primers ECF2_Fw and ECF2_Rv, ECF3_Fw and ECF3Rv, ECF5_Fw and ECF5Rv, were used to confirm the second crossover events.

#### Construction of a deletion mutant for ECF gene Sig04314 in Streptomyces sp. ADI 96 − 15

The vector for deletion of gene 04314 was constructed by PCR amplifications of regions flanking the gene, using primers Sig04314A-F, Sig04314A-R for A flank, and Sig04314B-F, Sig04314B-R for the B (Table S1, Supplementary Information). Flank A was then cut with *AgeI* + *Pst*I, flank B- with *Pst*I + *Xba*I. After digestion, fragments A and B were ligated together with the 7.3 kb fragment from pKNOCK, digested with *Age*I + *Xba*I. The final construct pSig04314 was introduced into the *Streptomyces* sp. ADI 96 − 15 by conjugation. First crossover mutant passed several rounds of growth in liquid TSB medium, and was plated for sporulation. The − 5 dilution of spore suspension was plated on 10 SFM plates, and colonies that did not produce indigoidine were chosen as second crossover candidates. Their genomic DNA was extracted and used for PCR to confirm the deletion of the gene using primers 04314delCheck-F and 04314delCheck-R (Table S1, Supplementary Information).

### RNA sequencing

Cultures grown in MYM medium for 18, 24 and 48 h past ethanol shock, or without one, were used to prepare samples for RNA sequencing according to Sekurova et al. [9]. RNA isolation, preparation of cDNA and sequencing were performed at LGC Genomics (Germany). All the sequencing data were deposited at NCBI under the BioProject PRJNA888945 (https://www.ncbi.nlm.nih.gov/bioproject/?term=PRJNA888945).

### Differential gene expression and bioinformatics-based protein analyses

Raw reads were cleaned with prinseq-lite v0.20.4 [13] using the following parameters: trim_qual_right 28, min_len 75, min_qual_mean 25. Cleaned reads are mapped to a reference genome of *S. venezuelae* NRRL B-65,442 (NZ_CP018074.1) using BWA-MEM v0.7.16a [14] with default parameters. Aligned reads were filtered with SAMtools sort v1.15.1 [15] and used to calculate feature counts using htseq-count of HTSeq v2.0.1 [16] with parameter nonunique = none. Contamination was estimated using unaligned reads (filtered with SAMtools) and Kraken2 v2.1.2 [17] with the standard 8GB Kraken 2 Database (built in 2019). Calling of differential expression was performed by EdgeR v3.36.0 [18] (TMM normalization method and quasi-likelihood F-test) and DESeq2 v1.34.0 [19] (default parameters), considering only genes with absolute fold change greater than 0.6 and P-value less than 0.5 differentially expressed. The results of both methods were combined on consensus principle, with genes only being classified as differentially expressed, if both methods agree.

Analysis of proteins for the presence of specific domains and transmembrane helices was carried out using online tools MOTIF Search (https://www.genome.jp/tools/motif/) and DeepTMHMM (https://dtu.biolib.com/DeepTMHMM), respectively.

### Pathway analysis for secondary and primary metabolism

Gene function and pathway presence were predicted with gapseq v1.1 [20], based on the reference genome of *S. venezuelae* strain NRRL B-65,442. Genes predicted by gapseq were matched with reference genome loci by location under the conditions of more than 95% sequence overlap and unique matching. Associations of genes to reactions and pathways were selected based on the following criteria: Either gene, reaction, pathway associations were present in the draft metabolic model generated by gapseq, or such associations had a blast bitscore greater than 200 and were assigned a “good_blast” label in the functional prediction step of gapseq.

These gene-pathway associations were combined with their differential gene expression status to conduct gene set enrichment analysis. The analysis was performed with the GSEApy package and enrichr [21].

### LC-MS analyses and data interpretation

The extracts from *S. venezuelae* NRRL B-65,442 wild-type and mutants were analyzed by an untargeted LC-MS method with an LTQ Orbitrap Velos mass spectrometer (Thermo Fisher Scientific) exactly as described previously [9]. The identification of the metabolites of interest is also reported in detail in Sekurova et al. [9]. The targeted comparison of the peak intensities between different samples or groups of samples was performed with Skyline 21.1.0.146 [22]. The statistical differences between groups were evaluated using either the two-way analysis of variance (ANOVA) followed by the Tukey’s multiple comparisons test or the one-way ANOVA followed by the Dunnett’s multiple comparisons test (using Prism 9.5.1; GraphPad Software).

LC-MS analyses of the MeOH extracts from *Streptomyces* sp. ADI96-15 wild-type and knock-out strain cultures were performed on a Vanquish Horizon UHPLC system (Thermo Fisher Scientific) coupled to the ESI source of a timsTOF fleX mass spectrometer (Bruker Daltonics). Separation was carried out on an Acquity Premier HSS T3 column, 2.1 × 150 mm, 1.8 μm (Waters) using water and acetonitrile/water 9:1, both modified with 0.1% formic acid, as mobile phase A and B, respectively. The sample components were separated and eluted with a gradient starting with a linear increase from 0 to 20% B in 10 min, followed by a linear increase from 20 to 100% B in 25 min, and finally an isocratic column cleaning (4 min at 100% B) and re-equilibration step (6 min at 0% B). The flow rate was 0.5 mL/min and the column oven temperature was set to 40 °C.

High-resolution ESI-MS and MS/MS spectra were recorded in positive ion mode in the range of *m/z* 100–2500. CID spectra of the five most intense precursor ions in each MS^1^ spectrum were obtained in automated data-dependent acquisition mode using nitrogen as collision gas. The sum formulas of the detected ions were determined using Bruker Compass DataAnalysis 5.3 based on the mass accuracy (Δm/z ≤ 5 ppm) and isotopic pattern matching (SmartFormula algorithm). Dereplication was accomplished with the aid of The Natural Products Atlas [23], GNPS Library Search [24], and CAS SciFinder (American Chemical Society). MZmine 3 was used for comparing peak intensities between different samples or groups of samples [25]. The peak areas of the dereplicated compounds were determined using Skyline 23.1.0.268 [22] and the statistical differences between the Δ*sig04314* mutant and wild-type of *Streptomyces* sp. ADI96-15 was evaluated using an unpaired t test assuming Gaussian data distribution using Prism 10.0.3 (GraphPad Software).

## Results

### Effect of the small ribosomally synthesized peptide PepX on the metabolome and transcriptome of *S. venezuelae*

**Fig. 1:**
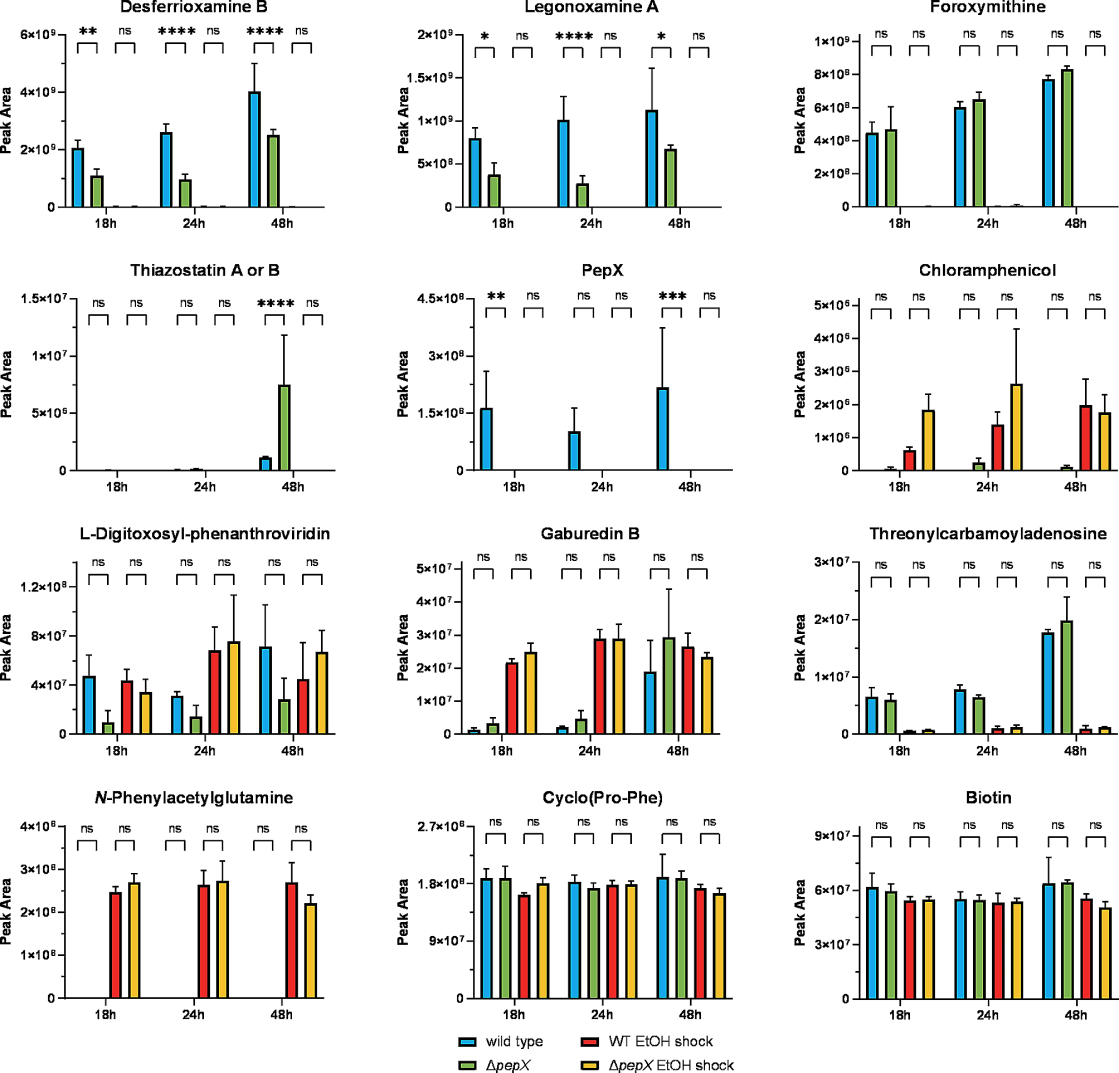
Effect of *pepX* deletion and ethanol shock on the production of secondary metabolites and the relative concentration of other identified compounds in MYM cultures of *S. venezuelae* NRRL B-65,442 after 18 h, 24 h, and 48 h of cultivation. After 7 h of cultivation, either 6% (v/v) of absolute ethanol (red and yellow bars) or distilled sterile water (blue and green bars) was added. Each bar represents the average of three independent experiments (*n* = 3). The error bars represent the standard deviation. The statistical differences between groups were evaluated using two-way ANOVA followed by Tukey’s multiple-comparison test (****, *P* < 0.0001; ***, *P* < 0.001; **, *P* < 0.01; *, *P* < 0.05)

In our previous study focused on the effect of ethanol shock on the transcriptome and secondary metabolome of *Streptomyces venezuelae*, we identified a conserved 19-amino acid peptide PepX, produced in copious amounts under normal conditions but drastically downregulated by ethanol shock [9]. Since this effect correlated with both up- and downregulation of certain BGCs, we hypothesized that PepX might play a role in controlling secondary metabolism. To test this hypothesis, we deleted “in frame” the *pepX* gene, leaving just the first and the last codons intact, and studied the resulting mutant regarding gene expression patterns and secondary metabolite biosynthesis with and without ethanol shock, comparing it with the wild type strain. Only a few differences in the secondary metabolomes of the wild type strain and the Δ*pepX* mutant could be detected (Fig. 1). In particular, the *pepX* deletion mutant showed a significantly lower production of the closely related hydroxamate siderophores desferrioxamine B and legonoxamine A, while levels of foroxymithine, another hydroxamate siderophore, were not altered. This effect was observed at all three time points (18, 24 and 48 h), but only in the absence of ethanol shock, which largely depletes these compounds in both the Δ*pepX* mutant and the wild type strains. Conversely, the concentrations of the 2-hydroxyphenylthiazoline congeners, such as thiazostatins A and B, were significantly higher after 48 h of cultivation of the Δ*pepX* mutant in the absence of ethanol shock. The biosyntheses of all other identified secondary metabolites were either not affected or the biological variation in their production levels was too high to enable detection of a statistically significant influence of the *pepX* deletion. The same holds true for all other metabolites previously shown to be up- or downregulated by the ethanol shock, such as threonylcarbamoyladenosine or *N*-phenylacetylglutamine, as well as for selected metabolites that were not affected by ethanol shock, such as diketopiperazines and biotin [9]. Clearly, at the level of secondary metabolites, the response to ethanol shock was nearly identical for the wild type strain and the Δ*pepX* mutant, ruling out the possibility that PepX is a major mediator of this effect.

In comparison to the wild type *S. venezuelae*, the Δ*pepX* mutant exhibited different responses to ethanol shock in terms of gene expression patterns. At the 18-hour time point post ethanol shock, 417 genes were downregulated and 354 were upregulated by ethanol shock in the Δ*pepX* mutant, while in the wild type strain these numbers were 431 and 679, respectively. It should be noted that there was a significant overlap between the differentially expressed genes in the mutant and the wild type subjected to ethanol shock, but it was not complete. The numbers of down/upregulated genes in the Δ*pepX* mutant were 349/308 and 327/213 at 24 h and 48 h post ethanol shock, respectively, while the corresponding numbers for the wild type strain were 496/579 and 308/305. It is worth noting that in the case of the Δ*pepX* mutant subjected to ethanol shock, the time-dependent decline in the number of downregulated genes was much steeper compared to the decline in upregulated genes. Thus, it appears that the effect of ethanol shock on gene expression in the Δ*pepX* mutant is milder relative to the wild type strain.

The RNAseq-based transcriptomics also revealed a significant effect of *pepX* deletion on gene expression patterns even in the absence of ethanol shock. Compared to the wild type strain, 224 genes were up- and 486 genes were downregulated in the Δ*pepX* mutant at 18 h time point, changing to 215 up/332 down at 24 h and 90 up/129 down at 48 h. According to the transcriptome data for 18 h, which revealed the most profound changes, genes within 7 BGCs (including those for desferrioxamines, jadomycins and foroxymithins) were downregulated and genes in 3 BGCs (including those for watasemycins and chloramphenicol) were upregulated in the Δ*pepX* mutant as compared to the wild type strain (Table 1). This largely fits the LC-MS data on cognate secondary metabolites, although the observed differences in the levels of L-digitoxosyl-phenanthroviridin (jadomycin precursor) and chloramphenicol did not reach a confidence level > 95% (Fig. 1). This picture to a certain extent resembled the response of the wild type strain to ethanol shock (15 BGCs down- and 3 upregulated). In particular, expression of genes for the biosynthesis of chloramphenicol (BGC9), desferrioxamine (BGC11), cryptic peptide-polyketide hybrid (BGC26), ladderane (BGC27) and foroxymithines (BGC33) was affected in the same way by the *pepX* deletion as by the ethanol shock in the wild type strain.

**Table 1:**
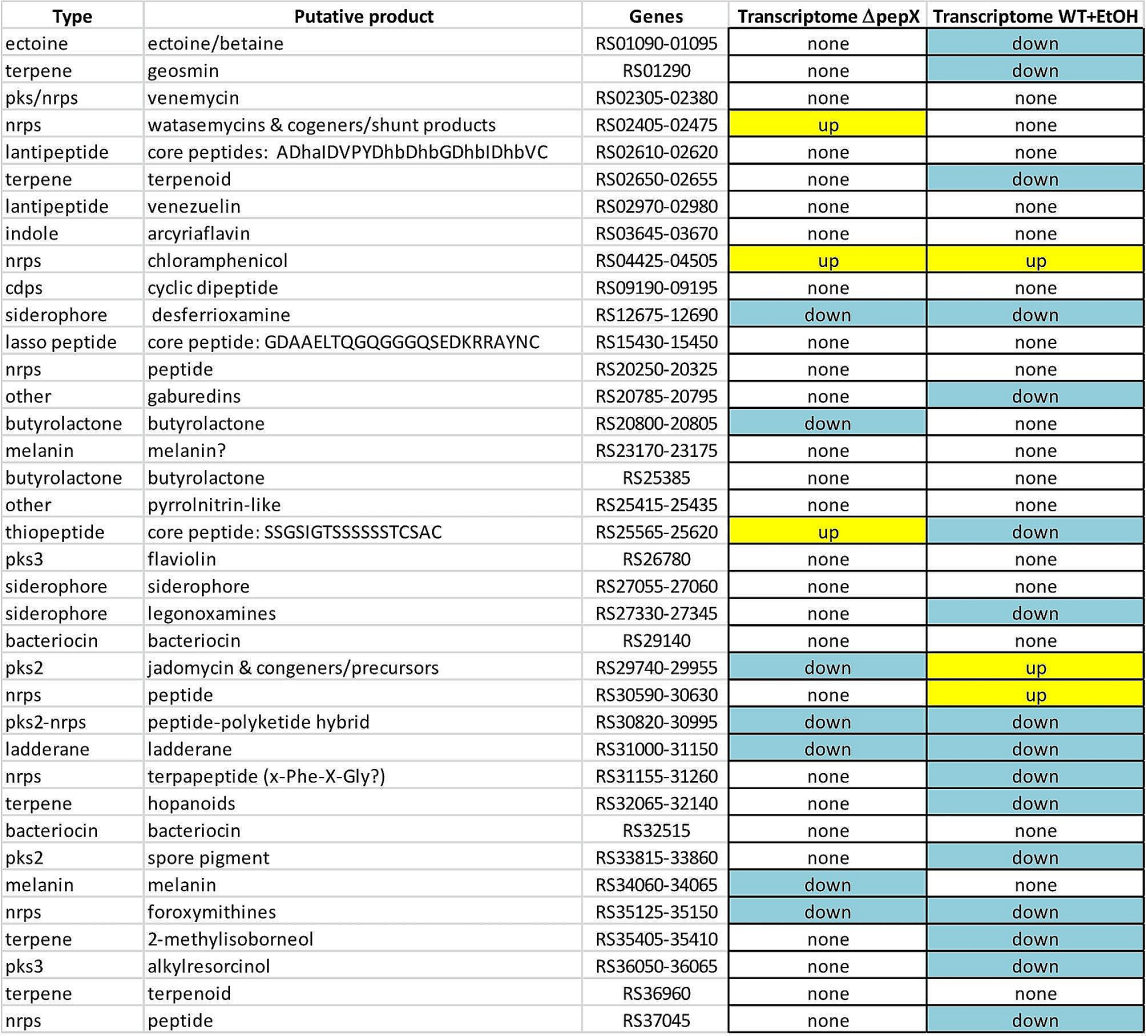
Comparison of the effects the *pepX* deletion and ethanol shock had on the BGC expression in *S. venezuelae*

Apart from the BGCs, we also analyzed the impact of the *pepX* deletion on the primary metabolism-related transcriptome. While most genes coding for enzymes in primary metabolism did not exhibit any significant change in expression, two pathways were strongly affected. In the arginine biosynthesis pathway, 3 and 4 genes were upregulated at the 18 h and 24 h time points, respectively, under conditions without ethanol shock. The products of all these genes are involved in the conversion of glutamate to ornithine. After ethanol shock, 4, 5 and 4 genes for enzymes of the glutamine biosynthesis pathway were found to be upregulated at 18 h, 24 h and 48 h, respectively. In addition, gene set enrichment analysis identified two primary metabolism pathways as significantly enriched in the set of differentially expressed genes of the Δ*pepX* mutant. These pathways are the pyridoxal 5’-phosphate (vitamin B6) biosynthesis and threonine degradation at 24 h and 48 h time points, respectively.

We have also performed an experiment on overexpression of *pepX* from a strong constitutive promoter P21 [11] in *S. venezuelae* using integrative vector (see Materials and Methods). The recombinant strain harboring the *pepX* overexpression vector pPXE1 along with the strain carrying empty vector pSOK806 [12] were analyzed for production of chloramphenicol, jadomycin-related metabolites, PepX itself, desferrioxamine, gaburedins and foroxymithin. This was done both with and without ethanol shock. The results of this experiment were rather inconclusive, since the vector alone had effect of the secondary metabolite production and standard deviations within three biological replicates were too high (see examples for gaburedins and jadomycin-related compounds in Fig. 4S, Supplemental Information).

### *pepX* deletion and ethanol shock effects on the expression of ECF-coding genes

Considering the partial overlap of the effects on secondary metabolism by the *pepX* deletion and ethanol shock, we explored the possibility that these effects may be mediated via ECF sigma factors. We examined the expression patterns of sigma factor-encoding genes in the Δ*pepX* mutant cultures at 18 h, 24 h and 48 h time points. Compared to the wild type strain, which has 56 sigma factor-coding genes, 12 such genes (10 of those encoding ECFs) were differentially regulated in the Δ*pepX* mutant (Table 2). Six of these genes, all encoding ECFs, were also affected in the wild type by the ethanol shock [9]. At the 18 h time point, 5 ECF-encoding genes and 2 genes for the housekeeping sigma factors were downregulated in the Δ*pepX* mutant, while the expression of only one ECF-coding gene was upregulated as compared to the wild type strain (Table 2). After 24 h, 2 ECF-coding genes and one for the housekeeping sigma factor were downregulated, while 3 ECF-coding genes were upregulated in the Δ*pepX* mutant. The effect of *pepX* deletion was much less pronounced after 48 h, with none of the sigma factors-coding genes being differentially expressed relative to the wild type.

**Table 2:** Expression of *S. venezuelae* σ factors affected by the deletion of the *pepX* gene relative to the wild type strain. Cells representing genes that encode ECFs are shaded in grey

Gene	18 h	24 h	48 h	σ domains
RS00060	-	Up	-	r2, r4
RS00830	Down	-	-	r2, r4
RS01725	Down	-	-	r2, r3, r4
RS15950	-	Up	-	r2, r4
RS16265	Up	-	-	r2, r4
RS17230	-	Up	-	r2, r4, SnoaL
RS18175	Down	Down	-	r2, r3, r4
RS18620	Down	Down	-	r2, r4
RS21030	Down	Down	-	r2, r4
RS22785	-	Up	-	r2, r4
RS23885	Down	-	-	r2, r4
RS34660	Down	-	-	r2, r4

The genes located immediately next to the sigma factor genes affected by the *pepX* deletion were analyzed for potential to encode anti-sigma factors (ASFs) or other proteins known to control ECFs [1]. ECF-encoding RS00060 gene is co-transcribed with RS00065, which codes for a 102 amino acid protein with one transmembrane domain predicted by DeepTMHMM software [26]. It seems plausible that this protein represents a membrane-embedded ASF of an unknown family. Putative transmembrane proteins that may be involved in the control of cognate ECFs were also identified for RS00830 and RS16265, while proteins with C_2_H_2_ zinc-finger domains known to sense redox potential and sequester ECFs [27] were found to be encoded next to RS18175, RS21030 and RS23885.

### Deletion of three ECF-encoding genes has profound differential effect on the primary metabolism and expression of *S. venezuelae* BGCs

Our previous data on the effect of ethanol shock on gene expression in the wild type strain [9] revealed three ECF-encoding genes, whose expression was upregulated at all three time points, namely RS15950, RS22785 and RS24280. Expression of two of these genes, RS15950 and RS22785, was also upregulated in the Δ*pepX* mutant without ethanol shock. Keeping in mind that most of the BGCs in *S. venezuelae* were downregulated by either ethanol shock or *pepX* deletion (Table 1), we reasoned that these particular ECFs might be involved in suppressing particular secondary metabolite biosynthesis pathways.

**Fig. 2:**
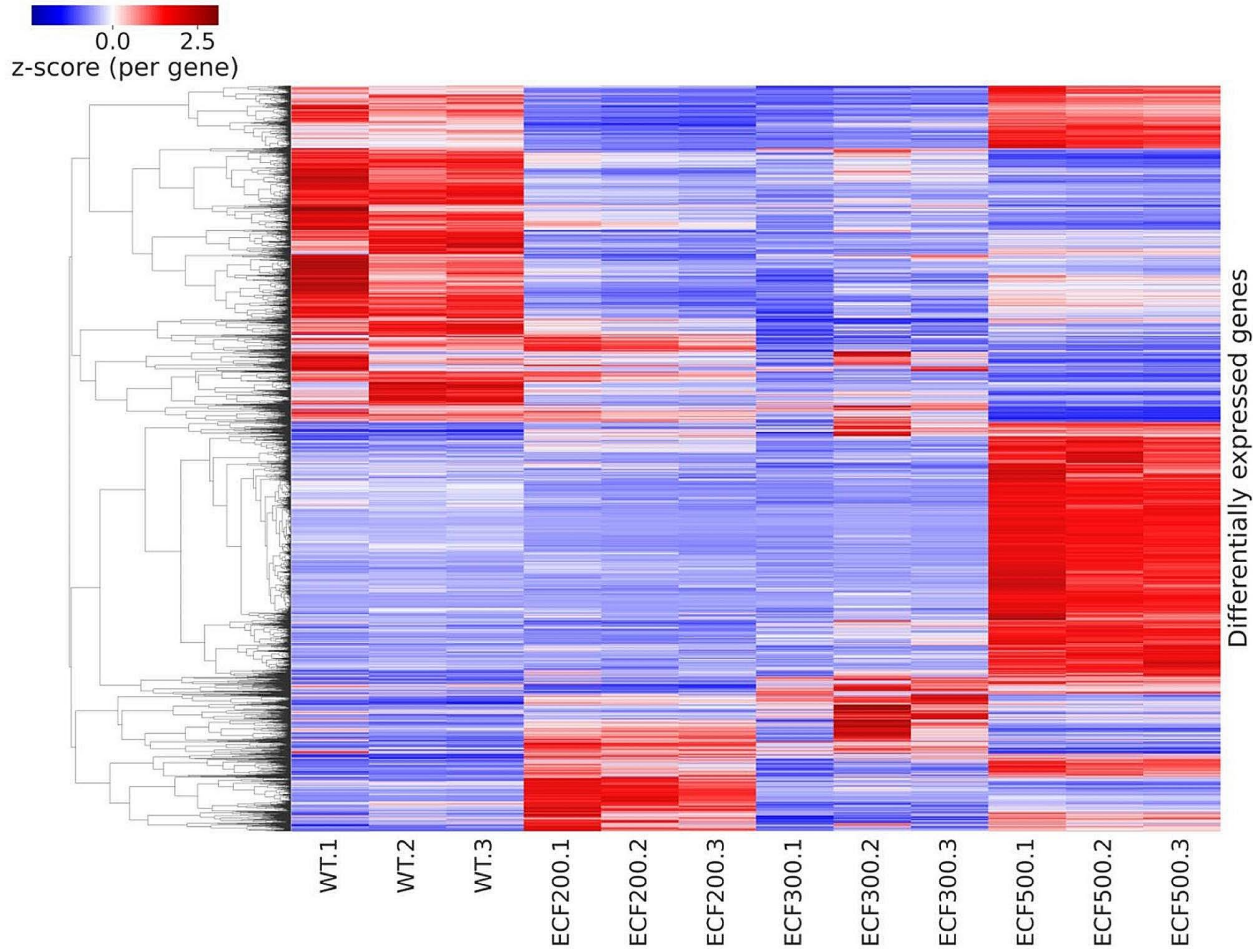
Comparison of differentially expressed genes among ECF mutants. The sample names show the genotype (wild type, ECF200, ECF300, ECF500) and the biological replicate [1–3]. Each cell in the heatmap corresponds to a gene in a given sample. The cells are colored with respect to gene expression, normalized within each sample to the overall expression and converted to a z-score based on mean and standard deviation across all samples. The order of the genes was determined by clustering, based on the z-score values

All three genes were individually deleted in the wild type strain, and the transcriptomes and targeted secondary metabolomes of the resulting mutants ECF200 (ΔRS15950), ECF300 (ΔRS22785) and ECF500 (ΔRS24280) were investigated. The heatmap showing gene expression patterns of the resulting mutants is shown in Fig. 2. Deletion of the ECF-coding gene RS15950 caused downregulation of 1433 genes (including 12 genes for sigma factors) and upregulation of 1511 genes (including 13 genes for sigma factors). Deletion of the ECF-coding gene RS22785 resulted in downregulation of 1309 genes (including 12 genes for sigma factors) and upregulation of 1433 genes (including 10 genes for sigma factors). Deletion of the ECF-coding gene RS24280 caused downregulation of 2187 genes (including 16 genes for sigma factors) and upregulation of 2366 genes (including 14 genes for sigma factors). The sigma factor-coding genes differentially affected in at least one of the three mutants (44 out of total 56) are shown in Table 3.

**Table 3:** Differential effect of ECF gene deletions on the expression of other sigma factor-encoding genes in *S. venezuelae*. Only genes affected in at least one of the mutants are shown

Sigma factor-encoding gene	Sigma factor domains	ΔRS15950	ΔRS22785	ΔRS24280
RS00270	r2, r4	NA	NA	up
**RS00630**	r2, r4, SnoaL	down	down	NA
**RS00830**	r2, r4	NA	NA	up
RS01100	r2, r3, r4	up	up	down
RS01725	r2, r3, r4	up	up	down
**RS01895**	r2, r4	NA	NA	up
RS02075	r2, CAP	NA	NA	up
RS02270	r2, r3, r4	NA	NA	up
**RS03595**	r2, r4, DUF6596	NA	down	down
**RS04145**	r2, r4, SnoaL	down	down	NA
**RS04775**	r2, r4	down	down	NA
**RS05705**	r2, r4	NA	NA	down
RS11135	r1, r2, r3, r4	up	NA	NA
RS13895	r2, r3, r4	down	down	down
**RS14430**	r2, r4	down	NA	NA
RS15140	r1, r2, r3, r4	down	down	down
**RS15760**	r2, r4	up	up	up
RS15920	r4, BACON	up	up	up
**RS15950**	r2, r4	**deleted**	up	NA
**RS16265**	r2, r4	NA	up	down
**RS16340**	r2, r4	down	NA	NA
**RS16715**	r2, r4	up	NA	NA
**RS17230**	r2, r4, SnoaL	up	up	up
**RS17255**	r2, r4, SnoaL	down	down	down
RS18175	r2, r3, r4	down	down	down
**RS18620**	r2, r4	NA	down	up
RS18755	r2, r3, r4	up	NA	down
**RS18935**	r2, r4, SnoaL	NA	NA	up
**RS19240**	r2, r4, NPCBM	NA	NA	up
**RS21030**	r2, r4	up	NA	NA
**RS22475**	r2, r4	NA	down	NA
**RS22610**	r2, r4	up	up	NA
**RS22720**	r2, r4, SnoaL	up	NA	NA
**RS22785**	r2, r4	**down**	**deleted**	**down**
**RS23885**	r2, r4	down	down	down
**RS24280**	r2, r4	NA	NA	**deleted**
RS24470	r2, r3, r4	up	up	down
**RS24785**	r2, r4	up	up	up
RS26440	r2, r3, r4	NA	NA	down
RS27450	r1, r2, r3, r4	NA	NA	down
**RS32930**	r2, r4	NA	NA	down
**RS33970**	r2, r4	NA	NA	up
RS34660	r2, r4	down	down	NA
RS35505	r2, r4, phage int	NA	NA	up

NA – not affected

### Effect of ECF gene deletions on the expression of genes involved in primary and secondary metabolism

The deletion of the three ECF genes also had a significant impact on gene expression related to enzymes in primary metabolism. Pathways for the biosynthesis of amino acids, as well as glycolysis, gluconeogenesis, fatty acid oxidation, and the TCA cycle were investigated in detail (see Supplementary Information, Table S3). On average, the fraction of differentially expressed genes in the pathways was close to the genome-wide prevalence. However, the distribution of up and downregulated genes in the pathways was not random (chi-square test, ECF300: p-value = 0.0289, ECF200 and ECF500: p-value < < 0,01). This is mainly due to the expression differences for genes involved in amino acid biosynthesis. In the ECF200 mutant more than 50% of genes were found to be upregulated in the pathways for arginine, asparagine, lysine, proline and serine biosyntheses, with only a single gene being downregulated in arginine biosynthesis. Conversely, in the case of the ECF500 mutant at least half of the genes in biosynthesis pathways for histidine, isoleucine, leucine, serine, threonine, tryptophane and valine were downregulated, with only one gene being upregulated in the tryptophane biosynthesis pathway. In addition, half of the genes in the pathways for glycine biosynthesis were upregulated, with none downregulated. The ECF300 mutant, however, had the fewest pathways with clear changes in the patterns of gene expression – only the alanine biosynthesis pathway is notable, with 2 genes being upregulated.

Next, we examined the effect of the three ECF genes deletions on the expression of the *S. venezuelae* BGCs in comparison with the wild type strain (Table 4). BGC expression was considered upregulated when more than one biosynthetic gene from the cluster was upregulated, and none of the other genes were downregulated. A similar rule was applied for considering downregulation of BGCs. This type of analysis still left some ambiguity with regard to the functional effect of gene expression patterns changes since in several BGCs, especially in the ECF200 mutant, some genes were up- and some downregulated. More specifically, 11 out of 37 BGCs in the ECF200 mutant were up- and 6 downregulated, with no effect observed for 8 BGCs. In the ECF300 mutant 11 out of 37 BGCs were up- and 13 downregulated, with no effect observed for 5 BGCs. The most profound effect on secondary metabolite biosynthesis genes expression was shown for the ECF500 mutant, where 14 BGCs out of 37 were up- and 8 downregulated, with no effect observed for only 2 BGCs.

**Table 4:** Effects of the three ECF-encoding gene deletions on the transcriptome and secondary metabolome of *S. venezuelae* as compared to the wild type strain

Putative product	Genes	Metabolome ΔECF200	Transcriptome ΔECF200	Metabolome ΔECF300	Transcriptome ΔECF300	Metabolome ΔECF500	Transcriptome ΔECF500
ectoine/betaine	RS01090-01095		up		up		none
geosmin	RS01290		up		up		up
venemycin	RS02305-02380		mixed		down		mixed
watasemycins & congeners/shunt products	RS02405-02475	none	mixed	up	mixed	up	mixed
core peptides: ADhaIDVPYDhbDhbGDhbIDhbVC;	RS02610-02620		up		down		mixed
terpenoid	RS02650-02655		up		up		down
venezuelin	RS02970-02980		up		none		up
arcyriaflavin	RS03645-03670		none		mixed		mixed
chloramphenicol	RS04425-04505	none	mixed	up	mixed	up	mixed
cyclic dipeptide	RS09190-09195		down		down		up
desferrioxamine	RS12675-12690	down	down	down	down	down	down
core peptide (?): GDAAELTQGQGGGQSEDKRRAYNC	RS15430-15450		mixed		up		mixed
peptide	RS20250-20325		none		up		up
gaburedins	RS20785-20795	up	none	up	up	up	up
butyrolactone	RS20800-20805		up		none		down
melanin?	RS23170-23175		up		down		down
butyrolactone	RS25385		none		none		none
pyrrolnitrin-like?	RS25415-25435		down		down		down
core peptide: SSGSIGTSSSSSSTCSAC	RS25565-25620		up		up		mixed
flaviolin	RS26780		none		none		up
siderophore	RS27055-27060		none		down		up
legonoxamines	RS27330-27345		mixed		mixed		mixed
bacteriocin	RS29140		none		down		up
jadomycin	RS29740-29955	up	mixed	up	mixed	none	mixed
peptide	RS30590-30630		mixed		mixed		mixed
peptide-polyketide hybrid	RS30820-30995		mixed		up		up
ladderane	RS31000-31150		mixed		mixed		mixed
terpapeptide (x-Phe-X-Gly? )	RS31155-31260		mixed		up		mixed
hopanoids	RS32065-32140		mixed		mixed		mixed
bacteriocin	RS32515		none		down		up
spore pigment	RS33815-33860		up		down		up
melanin	RS34060-34065		down		none		up
foroxymithines	RS35125-35150	up	mixed	up	down	up	down
2-methylisoborneol	RS35405-35410		up		up		up
alkylresorcinol	RS36050-36065		down		down		down
terpenoid	RS36960		down		down		down
peptide	RS37045		up		up		up

Expression of several BGCs was similarly affected by all three ECF gene deletions. In particular, in all three mutants expression of BGCs for geosmin, a cryptic lanthipeptide, a cryptic thiopeptide, flaviolin, 2-methylisoborneol and a cryptic non-ribosomally synthesized peptide was upregulated. Expression of four BGCs was downregulated in all three ECF mutants, namely those for desferrioxamine, a cryptic pyrrolnitrin-like compound, an alkylresorcinol and a cryptic terpenoid. Considering the eight sigma factor-encoding genes with similar changes in expression observed for all three mutants (Table 3), it is plausible that these regulate the expression of the abovementioned eleven BGCs either directly of via some kind of regulatory cascades.

Since several global regulators in *Streptomyces* are known to affect secondary metabolism as well as morphological differentiation, we examined expression of such genes in the ECF mutants. In particular, we looked at the expression of global positive regulator AdpA [28], Lsr2-type secondary metabolism repressors [29] and MtrA, which has been shown to play a differential role in controlling BGC expression [30, 31]. In addition, we took a closer look at the expression patterns of the regulatory genes in the chloramphenicol and jadomycin BGCs. The results of this analysis are presented in Table 5.

**Table 5:** Influence of the ECF gene deletions on the expression of known global and pathway-specific regulators in *S. venezuelae*

Gene	Streptomyces homologue	Role in regulation of secondary metabolism	ECF200	ECF300	ECF500
RS12725	AdpA	Activator [28]	-	down	down
RS16005	Lsr2	Repressor [34]	-	up	-
RS19015	Lsr2	Repressor [34]	down	-	down
RS13620	MtrA	Activator/repressor [30, 31]	-	-	down
RS04425	CmlR	Activator [29]/None [11]	down	down	down
RS29800	JadR1	Autoregulator [45]	down	down	down
RS29795	JadR2	Repressor [45]	down	down	down
RS29770	JadR3	Activator/repressor [46]	-	-	up
RS30005	JadR*	Repressor [47]	-	-	-

**Fig. 3:**
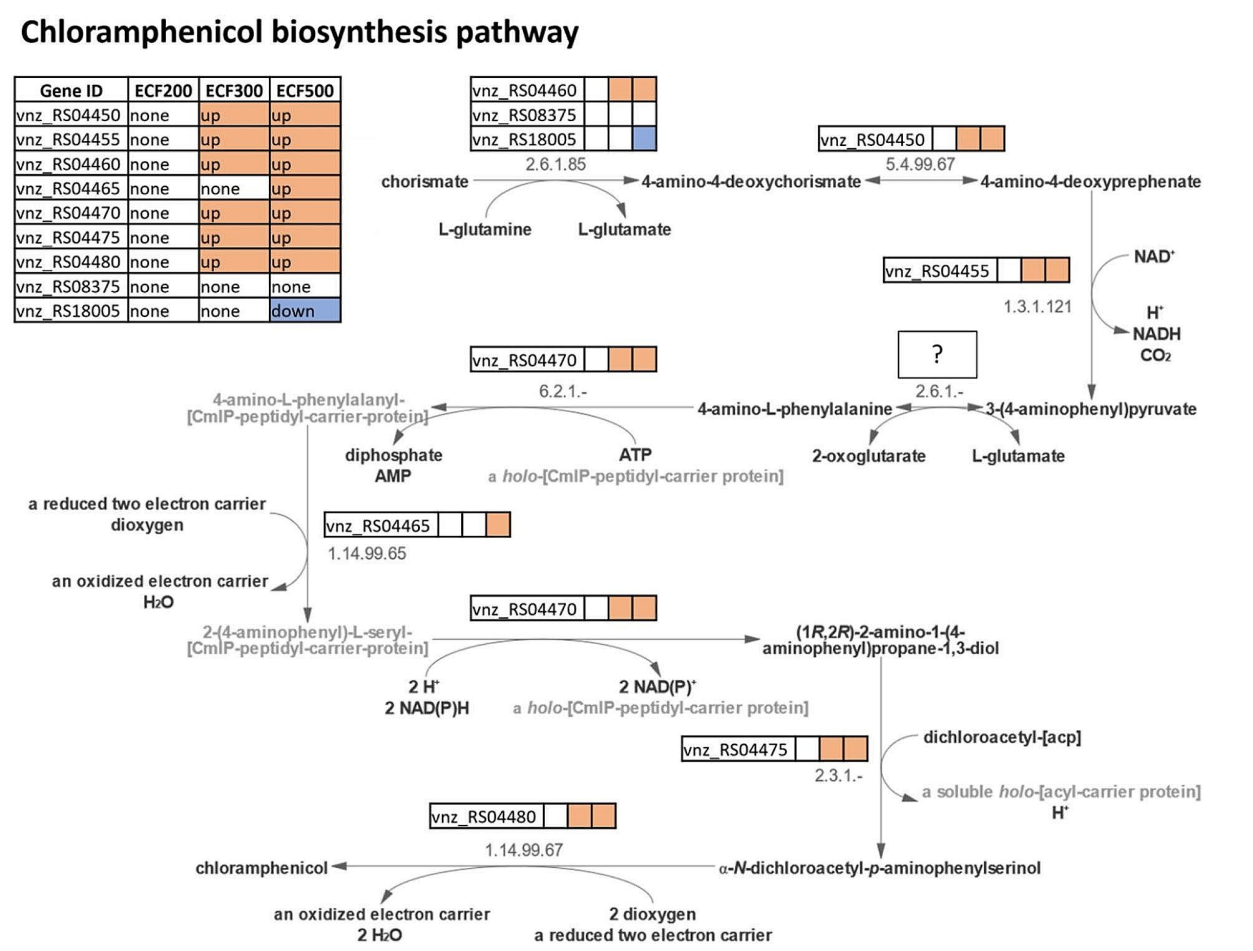
The chloramphenicol biosynthesis pathway visualized according to MetaCyc [37] (PWY-8032) and its gene expression in ECF deletion mutants. For each gene encoding an enzyme of this pathway the differential expression status is shown for the ECF200, ECF300 and ECF500 deletion mutants. Higher and lower differential expression is denoted as up and down in the table and color-coded with orange and blue respectively. For one step of the pathway, the conversion of 3-(4-aminophenyl)pyruvate to 4-amino-L-phenylalanine, no gene could be identified, marked here with a question mark

Although the correlation between the expression of global regulators and secondary metabolism in the ECF mutants was quite good (see Discussion), the same was not true in the case of the chloramphenicol BGC. The expression of the latter was a more complex case, as it includes both up- and down regulated genes in all three ECF gene deletion mutants. One of the genes downregulated in all the ECF mutants was RS04425, which codes for the CmlR protein shown to act as a positive regulator for chloramphenicol biosynthesis in *S. venezuelae* ATCC 10,712 [29]. The amino acid sequence of CmlR from ATCC 10,712 and NRRL B-65,442 differ in two amino acids, the latter having additional Pro_233_Gly_234_ in its C-terminus immediately downstream the DNA binding domain. This difference may account for the differences in how these two homologues function. It has been shown recently that chloramphenicol biosynthesis and its regulation differs remarkably between the strains of *S. venezuelae* [32]. Interestingly, 6 and 7 genes encoding enzymes for the chloramphenicol biosynthesis are upregulated in the ECF300 and ECF500 mutants, respectively, whereas only non-metabolic genes of this BGC are upregulated in the ECF200 mutant (Fig. 3). For the former two mutants, the reason for drastically increased chloramplenicol production are clear, since the genes encoding enzymes that are central in its biosynthetic pathway, namely CmlA, B, C, D, H, I and P are all upregulated. The most likely explanation for increase chloramphenicol production by the ECF200 mutant is increased supply of the chorismate, the starting metabolite crucial for its biosynthesis.

Among the many genes differentially expressed in the three ECF gene deletion mutants, gene set enrichment analysis found three pathways to be enriched: ectoine, UMP and meso-butanediol biosynthesis. All three pathways are enriched in both ECF200 and ECF300 mutants, but none in ECF500.

### Secondary metabolite profiles of the three ECF-encoding gene deletion mutants

**Fig. 4:**
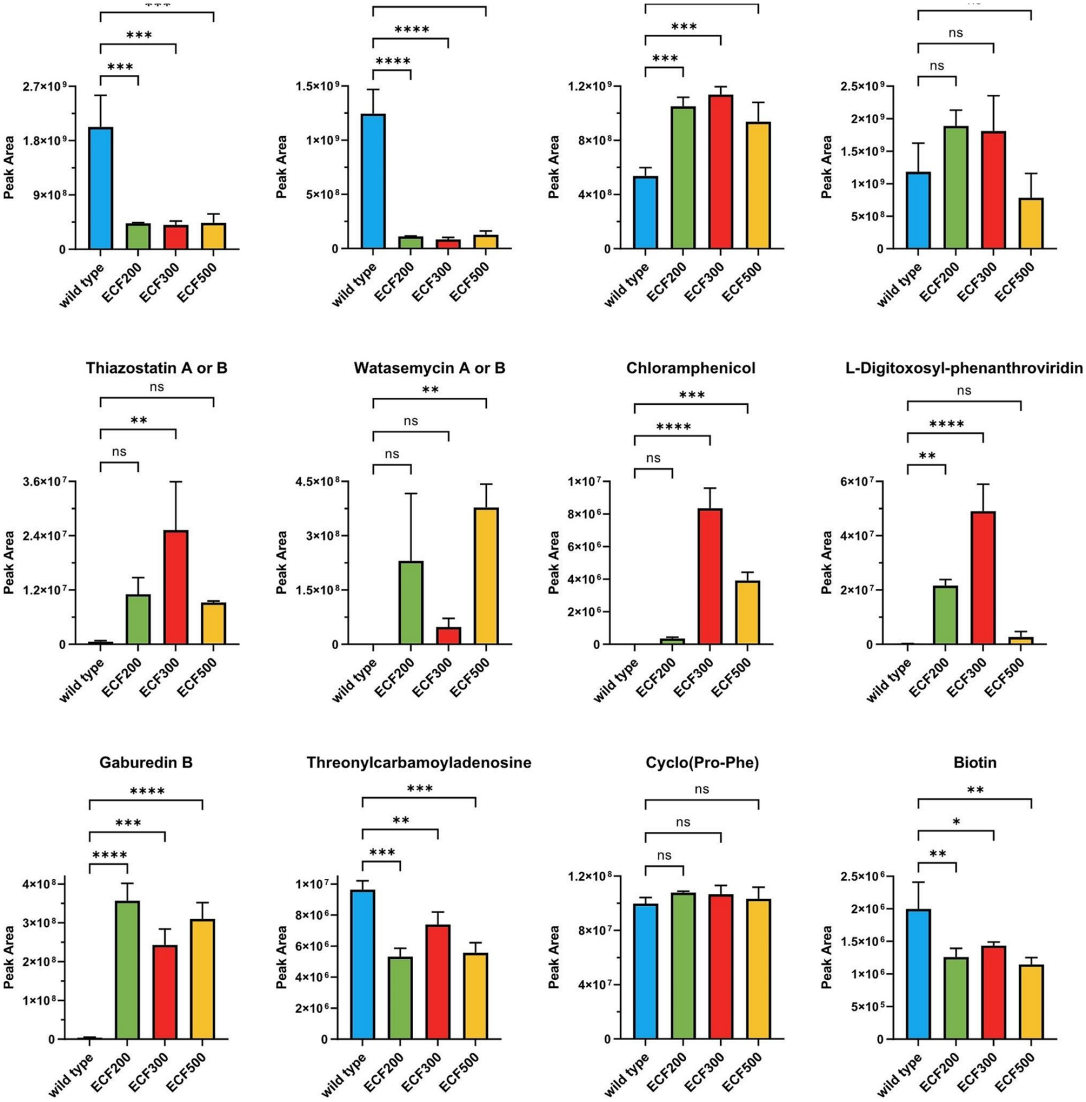
Effect of three ECF gene deletions on the production of secondary metabolites and the relative concentration of other identified compounds in MYM cultures of *S. venezuelae* NRRL B-65,442 after 72 h of cultivation. Each bar represents the average of three independent experiments (*n* = 3). The error bars represent the standard deviation. The statistical differences to the wild type were evaluated using one-way ANOVA followed by Dunnett’s multiple-comparison test (****, *P* < 0.0001; ***, *P* < 0.001; **, *P* < 0.01; *, *P* < 0.05)

The profound effect of the three ECF gene deletions on the expression of the *S. venezuelae* BGCs was also measurable on the metabolome level (Fig. 4). In agreement with the transcriptome data, a strong downregulation in the production of desferrioxamine B and legonoxamine A was observed. Conversely, higher concentrations of foroxymithine and particularly the gaburedins were found in the cultures of the mutants. While all the above-mentioned secondary metabolites were detected in very similar levels in the ECF200 (ΔRS15950), ECF300 (ΔRS22785) and ECF500 (ΔRS24280) mutants, a more diverse response was seen for the 2-hydroxyphenylthiazoline congeners, jadomycin-related angucyclines and chloramphenicol.

The concentrations of the watasemycin and thiazostatin congeners in the medium showed stronger batch-to-batch variations than those of the other secondary metabolites, limiting the confidence in the observed effects. However, they were consistently found in very low amounts only in the wild type, and a very strong increase in all three ECF-mutants is obvious. Interestingly, the data indicate relatively higher levels of thiazostatin A and B and relatively lower levels of watasemycin A and B in ECF300 compared to ECF200 and ECF500. The BGC required to produce these 2-hydroxyphenylthiazoline congeners is verified and several steps in the biosynthesis are well studied. The watasemycins, presumed to be the final products of the biosynthetic machinery, are obtained by *C*-methylation of the thiazostatins by a type B radical-SAM methylase [33]. The transcriptomics data do not show significant differences in the expression of the corresponding gene RS02465, which is the homologue of *sven0515* described for *S. venezuelae* ATCC 10,712 [33], but this would have to be studied in more detail, e.g. by time-resolved qPCR experiments. In general, no obvious correlation can be seen between the transcriptome and metabolome profiles regarding these compounds. However, the presence of three transcriptional regulators in the BGC and previous reports on the effects of regulatory proteins such as BldM [33] and Lsr2 [34] on the transcription of genes in this BGC suggest that the biosynthesis of thiazostatins and watasemycins is tightly controlled by a complex regulatory system.

Even more interesting from a biotechnological perspective is the tremendous increase in levels of the antibiotic chloramphenicol (up to 1700 fold, compared to the wild type) and the jadomycin-related angucycline L-digitoxosyl-phenanthroviridin shown particularly upon RS22785 deletion in ECF300. Interestingly, the gene expression profile of the enzymes involved in jadomycin biosynthesis shows an opposing pattern, with the majority of genes significantly higher expressed in the ECF500 mutant, but only a fraction of genes differentially expressed in the other two ECF mutants. The relevance of the effect on the concentration of L-digitoxosyl-phenanthroviridin is more difficult to evaluate since the baseline level in the wild-type shows strong fluctuations between batches and similar levels as in the ECF300 mutant were previously seen upon ethanol shock (Figs. 1 and [9]. However, the production of chloramphenicol in the ECF300 and ECF500 mutants was much higher than we have ever seen in the wild type, and in the case of ECF300 even surpasses the effect of ethanol shock. For comparison, constant levels of the diketopiperazine Cyclo(Pro-Phe) were measured, while a moderate but significant effect of all three ECF gene deletions on the concentrations of threonylcarbamoyladenosine and biotin were observed, indicating some wider alterations of the metabolism in line with the large number of up- and downregulated genes described above.

### Secondary metabolite profile of the sigma factor deletion mutant of *Streptomyces* sp. ADI96-15

The profound effect the deletions of the three selected ECF sigma factor genes had on secondary metabolome of *S. venezuelae*, and the fact that these ECFs are conserved in *Streptomyces*, prompted us to test whether similar results can be obtained in other *Streptomyces* species. As a target gene for deletion, the RS24280 homologue (which was deleted in the ECF500 mutant of *S. venezuelae*) was chosen, since this mutation, according to the RNAseq data, led to upregulation of the largest number of BGCs in *S. venezuelae*. For this experiment, we selected *Streptomyces* sp. ADI96-15, which was isolated from a marine sponge *Antho dichotoma* collected in the Trondheim fjord (Norway) [35]. Since extracts from this isolate did not display antimicrobial activities, the secondary metabolome of this ADI96-15 has not been investigated previously. Hence, the extracts from ADI96-15 culture grown in MYM medium were analyzed for the presence of known secondary metabolites. De-replication with LC-MS assisted by antiSMASH genome analysis led to the tentative identification of compounds derived from eight different BGCs. The most abundant congeners of each group were the hydroxamate siderophore desferrioxamine B, the cyclic octapeptide surugamide A [36], the γ-aminobutyrate urea gaburedin B, the class III lanthipeptide SAL-2242 [37], the polyene macrolide candicidin D [38] and a compound with the sum formula C_13_H_20_O_3_ that is most likely a γ-butyrolactone (GBL) as judged by the high similarity of its MS/MS spectrum to those of known GBLs (Table S1).

**Fig. 5:**
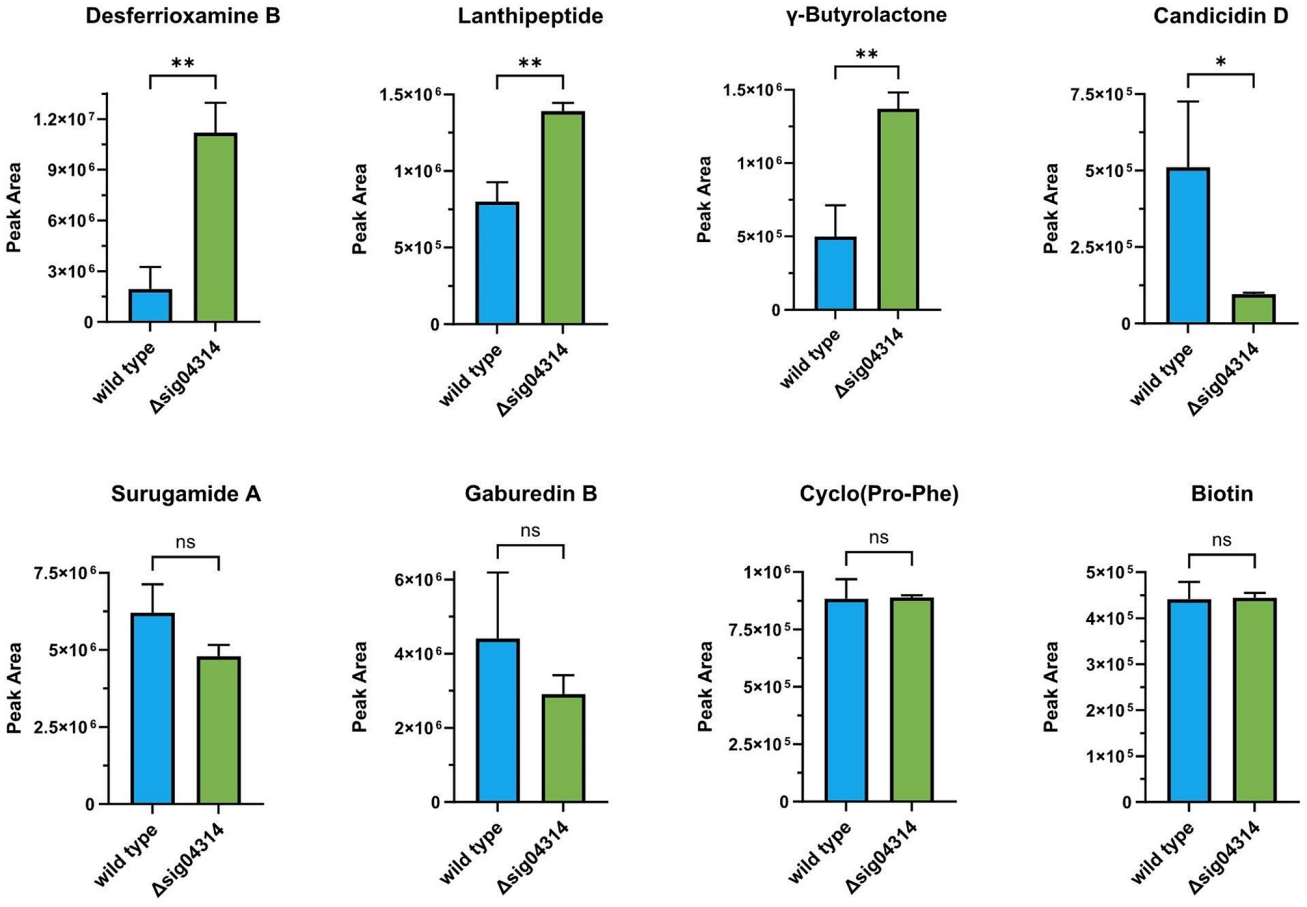
Effect of the RS24280-homologue deletion (*Δsig04314*) on the production of secondary metabolites and the relative concentration of other identified compounds in MYM cultures of *Streptomyces* sp. ADI96-15 after 7 d of cultivation. Each bar represents the average of three independent experiments (*n* = 3). The error bars represent the standard deviation. The statistical differences to the wild type were evaluated using an unpaired t test (**, *P* < 0.01; *, *P* < 0.05)

The homologue of the *S. venezuelae* gene encoding ECF500 (RS24280) was identified in the genome of ADI96-15 (GenBank accession number GCA_003846265.1) as str9615DRAFT_04314, later referred to as *sig04314*, its product showing 79% amino acid sequence identity to that of RS24280. *sig04314* was deleted from the chromosome of *Streptomyces* sp. ADI96-15 (see Materials and methods), and the secondary metabolome of the resulting mutant compared to that of the wild-type strain. As expected, the deletion of the *sig04314* gene in *Streptomyces* sp. ADI96-15 had a significant effect on the production of several secondary metabolites, thus confirming the role of this ECF in the regulation of natural product biosynthesis in this strain (Fig. 5). In contrast to our observations with the three ECF deletion mutants of *S. venezuelae* NRRL B-65,442, significantly higher amounts of desferrioxamine B were detected in the *Δsig04314* strain, while no significant effect was seen on the gaburedin B level. The production of the lanthipeptide SAL-2242 and the presumed GBL were also upregulated, while the one of candicidin D was downregulated. However, overall, the impact of the *sig04314* deletion was less pronounced than for any of the three ECF-coding gene deletions in *S. venezuelae* NRRL B-65,442, which can be due to the cultivation conditions used.

## Discussion

Our previous study on the effect of ethanol shock on gene expression in *S. venezuelae* [9] revealed certain interesting facts that required further investigation. In particular, a correlation between downregulation of certain secondary metabolite BGCs and expression of a *pepX* gene encoding a small, hydrophobic cryptic peptide with the sequence *N*-formyl-MNVITNLLAGVVHFLGWLV seemed intriguing. Although deletion of this gene had only modest effect on secondary metabolism, overall gene expression pattern was markedly changed in the Δ*pepX* mutant. Hence, *pepX* deletion appears to mimic the effect of ethanol shock to a certain extent, at least when it concerns particular secondary metabolite biosynthesis genes. If we consider changes in the bacterial membrane caused by ethanol [39], this observation may indicate that PepX is membrane-bound and plays a certain role as its component. The fact that only traces of PepX could be identified in the culture supernatant of the wild type strain (data not shown) indirectly supports this suggestion. Since PepX does not appear to have any transmembrane helices, it may be associated with the membrane via an interacting protein partner, and its absence affects membrane topology and hence membrane-bound proteins that control certain ECFs. Such interacting partner(s) might be encoded by the neighboring genes RS03775 and RS03780 annotated as coding for an endolytic transglycosylase of the YceG family and an ABC transporter, respectively. Endolytic transglycosylase functions as a peptidoglycan terminase that cleaves nascent peptidoglycan strands to terminate their elongation [40]. According to the prediction by DeepTMHMM and analysis by MOTIF Search online tool (https://www.genome.jp/tools/motif/), this protein has one N-terminal transmembrane domain with similarity to SLATT domain that has been implicated in cell signaling in bacteria [41]. The RS03780-encoded ABC transporter, according to the prediction, has six transmembrane domains and thus must be associated with the membrane. Notably, a similar genetic context in terms of genes surrounding *pepX* homologues were identified in many *Streptomyces*, e.g. *Streptomyces laurentii* ATCC 31,255, *Streptomyces fradiae* ATCC 10,745 and *Streptomyces coelicolor* M1154. In the genome of the latter strain, the *pepX* homologue is not annotated, but was identified between genes RS14905 and RS14910 coding for an endolytic transglycosylase and an ABC transporter, respectively. We have found copious amounts of PepX homologue in the extracts of *S. coelicolor* M1154 cultures (data not shown), supporting the suggestion that this small peptide must have a particular basic function in streptomycetes.

Inverse correlation between the expression of genes encoding three particular ECFs and certain BGCs in *S. venezuelae* prompted investigation into their effects on secondary metabolism by generating and analyzing deletion mutants. The high number of genes whose expression was affected in all three mutants, particularly in ECF500, is most likely due to a cascade-like effect because of the differential expression of other sigma factor-encoding genes (Table 2). Hence, establishing the true regulons of the three ECFs studied was not possible in this work, especially considering the possibility of spontaneous suppressor mutations [42].

The activity of ECFs in bacteria is usually controlled by specific anti-sigma factors (ASFs), which are often tethered to the membrane and sequester ECFs via protein-protein interactions. Alternatively, ECF activity can be modulated by soluble ASFs. In the case of *S. coelicolor*, σ^R^ is controlled by the soluble ASF RsrA that binds σ^R^ and invokes a conformational change that prevents this ECF’s binding to cognate promoters [43]. This interaction is broken by a change in redox conditions induced by environmental stimuli that prompt the formation of a disulfide bridge on RsrA, resulting in σ^R^ release. Out of three investigated ECFs of *S. venezuelae*, only RS24280 appears to have an ASF encoded by a downstream gene RS24281. This putative ASF is represented by a 105 amino acid protein predicted to contain a zinc-finger C_2_H_2_ domain. Such a domain is known to be involved in redox sensing [27], hence supporting the role of this ASF similar to that of RsrA in *S. coelicolor*.

How the ECF encoded by RS15950 is controlled is not entirely clear, as the latter gene is transcriptionally coupled to the downstream gene RS15955, which specifies a 254-amino acid protein with no homology to known ASFs or any other characterized proteins. Immediately downstream of RS15955, there are genes encoding a two-component system, which can be part of a control mechanism. Similarly, a two-component response system encoded by genes immediately upstream of RS22785 and transcribed in opposite direction may also control the latter ECF. The ECF σ^E^, a major regulator of cell envelope stress in *S. coelicolor*, is controlled by a two-component system [44], and it might be tempting to assume that ECFs responding to ethanol shock could play a similar role. However, this remains an open question, since no appreciable homology (< 30%) could be found between the amino acid sequences of σ^E^ and the three ECFs characterized in this study.

For a long time it has been assumed that sigma factors in general play a strictly positive regulatory role, as they are required components of a transcriptional complex essential for promoter recognition, formation of an open complex, and promoter clearance. Recent studies, however, challenge this paradigm, suggesting that ECFs can play both positive and negative roles in controlling gene expression, especially in *Streptomyces* bacteria. Rebets et al. [5] reported deletion of several sigma factor-encoding genes in *Streptomyces lividans* TK24, with some of the deletions apparently causing the activation of silent BGCs for actinorhodin and undecylprodigiosin in this strain. New investigation on the regulatory role of the σ^E^ ECF in *S. coelicolor* firmly established its role as both a positive and negative regulator [45], although whether the observed effects were direct or the results of a cascade-like regulation has not been studied. Our own data, showing that deletion of the three ECF-encoding genes in *S. venezuelae* positively affects the expression of particular BGCs, supports the differential negative regulatory roles of these ECF-encoding genes in secondary metabolism.

In this respect, it is worth noting the remarkable effect the ECF gene deletions had on the expression of regulatory genes reported to control relatively well characterized BGCs for chloramphenicol and jadomycin. The former BGC contains just one regulatory gene, CmlR, for which rather controversial data were obtained (see Results), suggesting that this cluster may be controlled by other, not yet identified activator or repressor. The regulation of jadomycin biosynthesis appears to be complex, and involves at least four cluster-situated regulatory genes. Genes for both autoregulator JadR1, described as a major transcriptional activator for jadomycin BGC and JadR2, the repressor controlling *jadR1* expression [46], are downregulated in all ECF deletion mutants. JadR3, which was shown to be SCB3 γ-butyrolactone-responsive repressor controlling expression of its own gene and *jadR2*, thus indirectly activates *jadR1* expression and was considered important for jadomycin biosynthesis [47]. While no effect on *jadR3* expression in both ECF200 and ECF300 mutants exhibiting highest level of jadomycin precursor production (Fig. 4) was observed, this gene was upregulated in the ECF500 mutant. The observed effects appear to reflect complex interactions within the regulatory network controlling jadomycin biosynthesis, which also includes jadR* gene that regulates co-factor supply [48] and was not affected in any of the mutants.

The fact that the deletion of RS24280 in ECF500 mutant had a positive effect on the expression of the majority of BGCs prompted us to test whether this effect can be mimicked by deleting a homologous ECF-encoding gene in another *Streptomyces*. We chose the marine sponge-derived *Streptomyces* sp. ADI96-15 because it is a natural isolate and a non-model strain with no previous record of its secondary metabolome. Deletion of the RS24280 homologue in this strain indeed had a significant effect on secondary metabolism, impacting at least four biosynthetic pathways for molecules that could be identified using LC-MS-based analytics. Notably, the effect of this deletion on desferrioxamine biosynthesis was opposite to that observed in *S. venezuelae*, suggesting a difference in regulation of this pathway in two *Streptomyces* strains. Considering these data, it appears that deletions of RS24280 homologues in *Streptomyces* spp. will deregulate secondary metabolism, while it is not possible to predict whether the effect on certain pathways will be positive or negative. This ambivalent effect seems logical, considering that differences in regulation of secondary metabolite biosynthesis pathways reflect exposure of particular strains to specific environmental cues. The three ECFs whose genes were shown in this study to be involved in regulation of secondary metabolism are conserved in hundreds of *Streptomyces* spp. This suggests that the deletions of homologous genes can be used as a tool to deregulate biosynthetic pathways, thereby eventually facilitating the discovery of new molecules with drug development potential or optimizing *Streptomyces* strains for improved secondary metabolite yield.

## Conclusion

*Streptomyces* bacteria represent a valuable source of bioactive secondary metabolites. However, often the genes involved in their biosynthesis remain transcriptionally silent under laboratory conditions, hindering the discovery of novel molecules. Additionally, yields of known valuable secondary metabolites can be low, posing significant challenges for their industrial production. In this study, we identified three ECF-encoding genes conserved in streptomycetes, whose individual deletions lead to differential upregulation of secondary metabolite biosynthesis genes and overproduction (up to 1700 fold) of certain cognate molecules. Therefore, deletion of homologous genes in various *Streptomyces* bacteria may offer a new effective tool for genome mining aimed at the discovery of new compounds, as well as for the increased production of known valuable metabolites.

### Electronic supplementary material

Below is the link to the electronic supplementary material.

Supplementary Material 1

## Data Availability

All the sequencing data generated during this project were deposited at NCBI under the BioProject PRJNA888945 (https://www.ncbi.nlm.nih.gov/bioproject/?term=PRJNA888945).
