# Supplementary material for: Deletions of conserved extracytoplasmic function sigma factors-encoding genes in Streptomyces have a major impact on secondary metabolism
Source: Microb Cell Fact. 2024 Jul 18;23:201. doi: 10.1186/s12934-024-02479-x (PMC11256431; doi:10.1186/s12934-024-02479-x)
Supplement: Supplementary file 1 — Supplementary Material 1 [file 12934_2024_2479_MOESM1_ESM.docx]

**Supplementary Information**

**Three conserved extracytoplasmic function sigma factors in *Streptomyces* have a major impact on secondary metabolism**

Olga N. Sekurova, Martin Zehl, Michael Predl, Peter Hunyadi, Thomas Rattei, Sergey B. Zotchev

**Table S1.** Oligonucleotide primers used for construction of gene deletion vectors.

| ***pepX* deletion** |  |
| --- | --- |
| PEXDF1 | CATGGAATTCTCGACGATCGGCGCCAA |
| PEXDR1 | CAGTGGATCCCTGGCCTCGTCGACGATCGC |
| PEXDF2 | CAGTGGATCCTCATGGTGGTGCTTCTCCTCG |
| PEXDR2 | GTCATCTAGAATGCCCGCGCTCATCTACTG |
| **ECF deletions** |  |
| ECF2delA-F | GATCTCTAGAGCCGATGGCGATCCTCATG |
| ECF2delA-R | GCTACTGCAGGTCGAGCACCTCGCCATG |
| ECF2delB-F | GACTCTGCAGGGAGAGCGGACGACCGTAC |
| ECF2delB-R | GTACGAATTCCTCGAGGAGCTTGTCACGG |
| ECF3delA-F | GATCCTGCAGCGTGACGAGGAGGAGTCCTTC |
| ECF3delA-R | GTACTCTAGAGTCCTCGTCCATGTGCAGC |
| ECF3delB-F | GATCCTGCAGGCTGTGCAGTGCGTTCATC |
| ECF3delB-R | GTACGAATTCCAGCGACTTGAACATGTAGC |
| ECF5delA-F | GTACCTGCAGAGAGTCGTCGAACGATCTGA |
| ECF5delA-R | GATCTCTAGAGAGCTTGCCGACATCGTG |
| ECF5delB-F | GCATCTGCAGCAGCACTGTCCAGTCGAG |
| ECF5delB-R | GTACGAATTCGAGGAGAAGCCCTCCTTCAAG |
| 04314A-F | GACGGAATTCGATCATCGAGCAGGAGGTC |
| 04314A-R | GATCCTGCAGACTTCAGCTGCTCTCCTCG |
| 04314B-F | GACTCTGCAGCTGCATCCTGCGGTCC |
| 04314B-R | GATCTCTAGACGTGATGTTCGAACAGTTCGC |

**Table S2**. ESI-QqTOF-MS data of the secondary metabolites produced by *Streptomyces* sp. ADI96-15 after 7 d of cultivation in MYM medium.

| R_t_ [min] | *m/z* [M+H]^+^ | [M+2H]^2+^ | sum formula (proposed) | *m/z*  (calcd.) | Δ*m/z* [ppm] | tentative ID |
| --- | --- | --- | --- | --- | --- | --- |
| 6.5 | 261.1444 |  | C_11_H_20_N_2_O_5_ | 261.1445 | 0.3 | Gaburedin B |
| 9.4 | 561.3605 |  | C_25_H_48_N_6_O_8_ | 561.3606 | 0.3 | Desferrioxamine B |
| 18.7 | 225.1484 |  | C_13_H_20_O_3_ | 225.1485 | 0.4 | Unidentified γ-butyrolactone |
| 21.0 |  | 1122.0567 | C_100_H_159_N_23_O_31_S_2_ | 1122.0580 | 1.1 | Lanthipeptide SAL-2242 |
| 21.0 |  | 555.2929 | C_59_H_84_N_2_O_18_ | 555.2932 | 0.5 | Candicidin D |
| 21.6 | 912.6277 |  | C_48_H_81_N_9_O_8_ | 912.6281 | 0.4 | Surugamide A or Champacyclin |

**Table S3**. Differentially expressed genes in primary metabolism pathways of the three ECF mutants.

|  | | **ECF200** | | | **ECF300** | | | **ECF500** | | |
| --- | --- | --- | --- | --- | --- | --- | --- | --- | --- | --- |
| **Pathway** | **total** | **down** | **up** | **deg%** | **down** | **up** | **deg%** | **down** | **up** | **deg%** |
| Alanine | 2 | 0 | 1 | 50% | 0 | 2 | 100% | 1 | 1 | 100% |
| Arginine | 15 | 1 | 8 | 60% | 2 | 3 | 33% | 4 | 2 | 40% |
| Asparagine | 2 | 0 | 2 | 100% | 0 | 1 | 50% | 0 | 1 | 50% |
| Aspartate | 17 | 2 | 3 | 29% | 5 | 4 | 53% | 4 | 7 | 65% |
| Cysteine | 6 | 1 | 1 | 33% | 1 | 1 | 33% | 2 | 3 | 83% |
| Glutamine | 12 | 4 | 2 | 50% | 3 | 3 | 50% | 5 | 2 | 58% |
| Glycine | 10 | 2 | 3 | 50% | 2 | 1 | 30% | 0 | 5 | 50% |
| Histidine | 10 | 1 | 1 | 20% | 0 | 0 | 0% | 5 | 0 | 50% |
| Isoleucine | 10 | 3 | 1 | 40% | 1 | 1 | 20% | 6 | 0 | 60% |
| Leucine | 5 | 0 | 0 | 0% | 1 | 3 | 80% | 3 | 0 | 60% |
| Lysine | 14 | 0 | 8 | 57% | 1 | 6 | 50% | 4 | 2 | 43% |
| Methionine | 10 | 2 | 2 | 40% | 2 | 1 | 30% | 3 | 4 | 70% |
| Phenylalanine | 18 | 2 | 3 | 28% | 5 | 4 | 50% | 5 | 7 | 67% |
| Proline | 7 | 0 | 5 | 71% | 0 | 3 | 43% | 1 | 3 | 57% |
| Serine | 3 | 0 | 2 | 67% | 0 | 0 | 0% | 3 | 0 | 100% |
| Threonine | 2 | 0 | 1 | 50% | 0 | 0 | 0% | 1 | 0 | 50% |
| Tryptophan | 10 | 3 | 0 | 30% | 2 | 1 | 30% | 6 | 1 | 70% |
| Tyrosine | 19 | 2 | 3 | 26% | 5 | 4 | 47% | 5 | 7 | 63% |
| Valine | 9 | 3 | 1 | 44% | 1 | 1 | 22% | 6 | 0 | 67% |
| TCA | 22 | 4 | 5 | 41% | 4 | 5 | 41% | 6 | 8 | 64% |
| Glycolysis | 15 | 2 | 2 | 27% | 3 | 3 | 40% | 7 | 3 | 67% |
| Gluconeogenesis | 14 | 4 | 3 | 50% | 5 | 4 | 64% | 6 | 3 | 64% |
| Fatty acid oxidation | 26 | 4 | 1 | 19% | 5 | 3 | 31% | 10 | 6 | 62% |
|  |  |  |  |  |  |  |  |  |  |  |
| Average (all) | 258 | 40 | 58 | 38% | 48 | 54 | 40% | 93 | 65 | 61% |
|  |  |  |  |  |  |  |  |  |  |  |
| Average (Amino acid) | 181 | 26 | 47 | 40% | 31 | 39 | 39% | 64 | 45 | 60% |

**Figure S1.**

**Figure S2.**

**Figure S3.**

**Figure S4.** Production of secondary metabolites by *S. venezuelae* overexpressing *pepX* gene in comparison with the strain harboring empty vector. (+) and (-) mean with or without ethanol shock, respectively.
